# Safety and effectiveness of rehabilitation training for stroke complicated with muscular call vein thrombosis: An observational study

**DOI:** 10.1097/MD.0000000000034574

**Published:** 2023-08-11

**Authors:** Benling Liu, Dong-mei Gao, Wen-han An, Fan-shuo Zeng, Bao-juan Cui, Laigang Huang

**Affiliations:** a Rehabilitation Medical Center, the Second Hospital of Shandong University, Cheeloo College of Medicine, Shandong University, Jinan, China.

**Keywords:** Fugl-Meyer Assessment score, muscular call vein thrombosis, stroke

## Abstract

This study aimed to explore the safety and effectiveness of rehabilitation treatment for stroke patients with muscular call vein thrombosis (MCVT) in the lower limbs. A total of 173 patients were recruited with stroke complicated by MCVT, including 130 who received rehabilitation training and 43 who did not receive rehabilitation training. The *t* test and chi-square test were used to analyze the basic data of the 2 groups. There were no significant differences in the Fugl-Meyer Assessment scores between 2 groups at the beginning of recruitment (*P* = .149). There was a significant difference in the Fugl-Meyer Assessment scores of the lower limbs in patients with MCVT after 3 weeks of rehabilitation treatment (*P* < .001), and there was a significant difference in the rate of MCVT recanalization and extension between the 2 groups (χ^2^ = 11.646, *P* = 0001). Combined with anticoagulation therapy, rehabilitation training did not increase the thrombosis progression of MCVT and was effective in the recovery of lower limb motor function in stroke patients.

## 1. Introduction

Stroke has a high incidence and disability rate.^[1]^ It is the first cause of disability in adults in China, and 70% to 80% of patients who have a stroke cannot live independently due to disability.^[2]^ Walking disorders are among the most important clinical dysfunctions following a stroke. Rapid improvement in lower limb function is one of the most important rehabilitation goals for patients.^[3]^

However, patients with acute stroke might have an increased risk of deep venous thrombosis (DVT) due to immobility, but most of them are asymptomatic^[4]^; therefore, it is uncertain when to intervene in rehabilitation treatment early, which will affect the prognosis of patients.

DVT of the lower limbs refers to abnormal coagulation and thrombosis of blood in the deep veins of the lower limbs caused by various reasons. The most common clinical type is muscular call vein thrombosis (MCVT), which refers to thrombosis occurring in the venous plexus of the calf gastrocnemius and soleus muscle, and belongs to the peripheral type of deep venous thrombosis, accounting for 30% to 50%.^[5]^ Kearon et al^[6]^ pointed out that patients with DVT in tolerable conditions should get out of bed early under the condition of effective anticoagulant therapy. At present, there are few studies on the safety of early rehabilitation training in stroke patients with MCVT. The author conducted a retrospective study of stroke patients in the rehabilitation department of our hospital to explore the safety and effectiveness of early rehabilitation training for stroke patients with MCVT.

## 2. Methods

This was a single-center retrospective study conducted at the Rehabilitation Medical Center of the Second Hospital of Shandong University. All inpatients signed informed consent forms, and no privacy was revealed.

**Figure 1.**
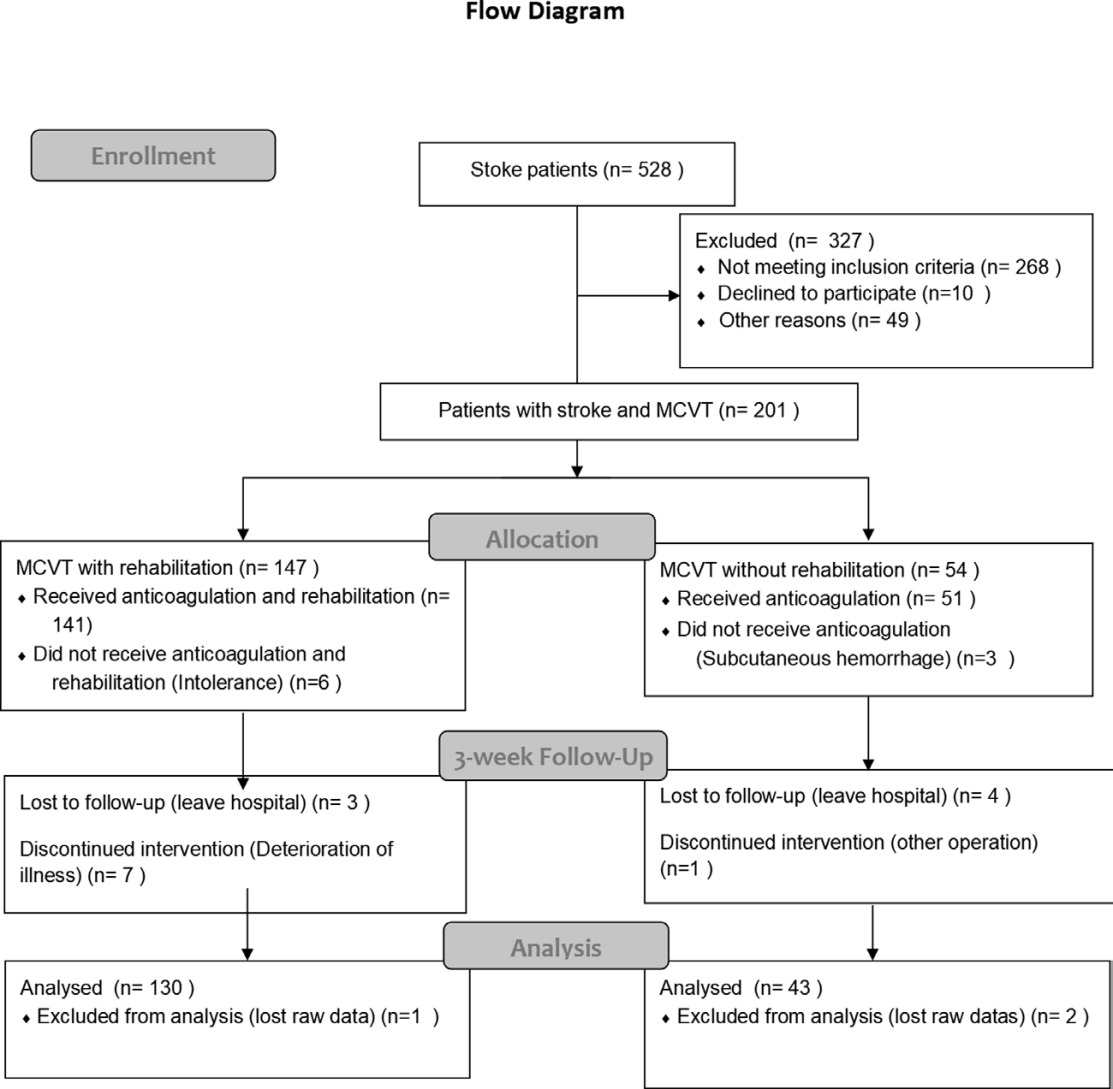
The flow chat of this study. MCVT = muscular call vein thrombosis.

A consecutive series of 528 patients was enrolled between September 2019 and March 2022. All patients` calves and proximal deep were evaluated using ultrasonography during the patient stay period. The flow chart is listed in Figure 1. The inclusion criteria were as follows.

Signs and symptoms of nerve defects (such as altered consciousness, paralysis, aphasia, dysphagia, ataxia, and paresthesia); computed tomography and/or magnetic resonance imaging performed after the onset of stroke; no previous stroke history; vascular ultrasonography performed on the paralyzed lower extremity or both lower-extremities within 2 weeks (ranging from 10–14 days) after the onset of stroke, regardless of whether the patient’s leg was swelling/painful or not^[4]^; and age between 13 and 90 years.

The exclusion criteria were as follows: recurrent stroke, history of fracture in the lower extremities, disability in the lower extremities before stroke, history of lower-extremity DVT prior to stroke onset and DVT in the paralyzed lower extremities, and severe infection and neoplastic diseases in the lower extremities.

### 2.1. Treatment

All patients were treated with enoxaparin 1 mg/kg subcutaneously once daily for 3 weeks at the time of diagnosis. A total of 173 patients completed this retrospective study, of which 130 patients received additional rehabilitation training, while 43 patients (non-rehabilitation group) refused rehabilitation training, which included acupuncture, neuromuscular electrical stimulation^[2]^and intensive exercises, a total of 120 minutes per day, 6 days per week.

During the entire follow-up, in the case of worsening symptoms and/or suspected DVT extension, patients were recommended to undergo a whole-leg DUS and Patients with symptoms of pulmonary embolism underwent diagnostic testing based on a multi-detector computed tomography scan.

### 2.2. Data collection

All information collected, including patients’ names, stroke categories (ischemic or hemorrhagic stroke), sex, age, and changes in lower extremity motor control from admission to 3 weeks, assessed by the Fugl-Meyer Assessment (FMA) scores, recanalization, and extension of the were retrospectively analyzed. CMVT recanalization was defined as increased blood flow or decreased thrombus on ultrasonography. Extention^[7]^ was defined as a decreased thrombus, proximal DVT, or pulmonary embolism^[8]^(PE) occurring within3week confirmed ultrasonography.

All clinical data were extracted and recorded from the electronic history and PACS system. The average hospital stay period of our department was 25 days, and 3 weeks was selected as the statistical node. The difference in lower limb motor function, assessed by the FMA score at admission and at 3 weeks, was calculated.

### 2.3. Statistical analysis

The primary outcome measure was the rate of MCVT recanalization/extension at admission and 3 weeks of treatment. The secondary outcome was the FMA score for lower extremity motor control from admission to 3 weeks.

Statistical analysis was carried out using SPSS software (version 26.0; IBM Corp., Armonk, NY). Continuous variables were expressed as mean ± standard deviation (SD). Continuous variables were compared using an independent sample *t* test and categorical variables were compared using the chi-squared test. Probability (*P*) values of .05 were considered statistically significant.

## 3. Results

### 3.1. Demographic characteristics

**Figure 2.**
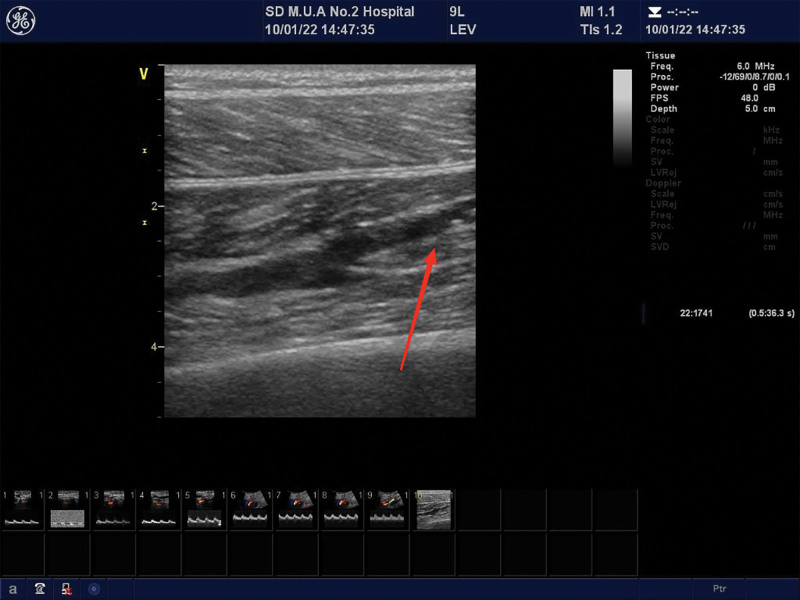
Visualized representation of MCVT in 2 patients were listed in Figure 2. The Red Arrows indicate MCVT which was diagnosed by vascular ultrasonography. MCVT = muscular call vein thrombosis.

A consecutive series of 173 patients were diagnosed with stroke and MCVT using vascular ultrasonography for DVT screening at the Center of Rehabilitation. The visualized representations of the MCVT are listed in Figure 2.

Patients in the Rehabilitation group comprised 88 male and 42 female patients, and the average age of these patients was 64.64 ± 9.56 years old (range:39–85 years old). Among these patients, 71 were diagnosed with ischemic stroke and 59 were diagnosed with hemorrhagic stroke.

And patients in the non-rehabilitation group were comprised of 31 male and 12 female patients, and the average age of these patients was 62.07 ± 10.27 years old (range: 45–84 years old). Among these patients, 25 were diagnosed with ischemic stroke, while 18 were diagnosed with hemorrhagic stroke.

Statistical analysis revealed no significant differences in age, sex and stroke types between the 2 groups (*P* = .136; χ^2^ = 0.291, *P* = .589; χ^2^ = 0.162, *P* = .687, respectively). The lower extremity FMA scores in the 2 groups were 4.34 ± 1.47 and 4.72 ± 1.59, respectively. No significant difference was observed (*P* = .149). The characteristics of the population are summarized in Table 1.

**Table 1:** Characteristics of the study population (x̅ ±s).

	MCVT with rehabilitation (n = 130)	MCVT without rehabilitation (n = 43)	χ^2^	*P*
Age	64.64 ± 9.56 (39–85)	62.07 ± 10.27 (45–84)	–	0.136
Sex				
Male	88	31		
Female	42	12	0.291	0.589
Stroke type				
Ischemic stroke	71	25		
Hemorrhagic stroke	59	18	0.162	0.687
FMA score（x̅ ±s）	4.34 ± 1.47	4.72 ± 1.59	–	0.149

FMA = Fugl-Meyer Assessment, MCVT = muscular call vein thrombosis.

### 3.2. Safety and effectiveness

Among the 130 patients receiving therapeutic anticoagulation and rehabilitation training, recanalization occurred in123 (94.62%) and 33 (76.74%) patients, respectively. Proximal DVT or PE occurred in 7 rehabilitation group patients (5.38%) and 10 non-rehabilitation patients (23.26%). The statistical analysis revealed significant differences (χ^2^ = 11.646, *P*^a^ = .001).

Proximal DVT was found in the popliteal (n = 2), posterior tibial (n = 6), and femoral veins (n = 3). Patients with PE were diagnosed using chest computed tomography, and no bleeding or death occurred. Therapeutic anticoagulation combined with rehabilitation training was associated with an increased likelihood of recanalization and a reduced likelihood of a composite of proximal DVT and PE.

Lower extremity FMA score 17.05 ± 2.39 in the rehabilitation group and 12.26 ± 2.98 in the non-rehabilitation group. The statistical analysis revealed significant differences (*P*^b^ < .001). Rehabilitation admission was significantly positively correlated with the lower extremity FMA score, which indicates a higher likelihood of effectiveness of rehabilitation services for stroke patients. The outcomes and FMA score at 3weeks are listed in Table 2.

**Table 2:** Outcomes and FMA score at 3 weeks.

	MCVT with rehabilitation No. (%)(n = 130)	MCVT without rehabilitation No. (%) (n = 47)
Recanalization	123 (94.62%)	33 (76.74%)^a^
Extension	7 (5.38%)	10 (23.26%)
Proximal DVT	5	7
PE	2	3
Lower extremity FMA score (*x̅*±s)	17.05 ± 2.39	12.26 ± 2.98^b^

χ^2^ = 11.646, *P*^a^ = .001; *P*^b^ < .001.

DVT = deep venous thrombosis, FMA = Fugl-Meyer Assessment, MCVT = muscular call vein thrombosis, PE = pulmonary embolism.

## 4. Discussion

Stroke primarily includes infarcts and hemorrhagic strokes. DVT and other complications are often caused by lower-limb paralysis, application of dehydrators, and other factors. Lower-extremity deep venous thrombosis (DVT) is a common complication in stroke patients with hemiplegia. The incidence rate can reach 3% to 30%, even as high as 53%.^[9]^

A higher incidence of DVT is common in patients with stroke paralysis. Furthermore, some researchers suggest that this difference may be caused by the administration of hypertonic dehydrating agents in hemorrhagic stroke, which can lead to hemoconcentration and an increase in blood viscosity.^[10]^

All patients with stroke should receive rehabilitation therapy as early as possible once they are determined to be rehabilitated and medically able to participate in active rehabilitation (Evidence Level A)^[2]^ within an active and complex stimulating environment. Evidence-based medicine confirms that early rehabilitation training is recommended for patients with subacute stroke, including leaving the bed as soon as possible,^[11]^ and standing, walking, and balance training.^[12]^

Patients should receive the recommended 3 hours per day of direct task-specific therapy, 6 days per week, delivered by the inter-professional stroke team. More therapy results in better outcomes (Evidence Level A).^[13]^

DVT may occur in the anterior tibia, posterior tibia, peroneal vein, and/or the common femoral vein.^[14]^ MCVT refers to DVT in the intermuscular venous plexus, which refers to a primary thrombosis confined to the gastrocnemius and soleus venous plexus, characterized by a slender caliber, abundant branches, fewer venous valves, interwoven venous network, absence of surrounding deep fascia, and slow blood flow.^[11]^

MCVT can spread to the deep vein and form a more serious deep vein thrombosis. It is also a source of PE, which cannot be ignored.^[15]^

Since DVT is an important risk factor for PE and sudden death, treatment of deep vein thrombosis remains a challenging problem. DVT can potentially lead to life-threatening complications,^[16]^ such as PE and recurrent DVT, with significant social and economic impact. Therefore, Safe and effective treatments are essential.^[17]^

Anticoagulation is the basic treatment for all types of lower extremity deep venous thrombosis. Its role is to inhibit the spread of thrombosis, facilitate thrombolysis and lumen recanalization, and reduce the incidence and mortality of pulmonary embolism. The recanalization rate of intermuscular venous thrombosis significantly increases after therapeutic anticoagulation (TA).^[18]^ Moreover, a previous study^[19]^ in similar patients who received a full dose of low molecular weight heparin and a preliminary report on a randomized trial, showed that heparin at a therapeutic dose for 2 to 6 weeks was an effective and safe alternative to warfarin in patients with MCVT.^[20]^ The results suggest the safety of the treatment protocol in all the patients, and during follow up, no major bleeding was observed.

The shrinking and recanalization rate of MCVT in the rehabilitation group was approximately 86.05% at 3 weeks after the diagnosis of CDVT and was not significantly different from that in the non-rehabilitation group. Similarly, a study reported a recanalization rate of 50% for 115 muscular DVT at 4 weeks.^[21]^ Guidelines^[22]^ suggest a 12-week treatment regimen for MCVT.^[22]^

In our hospital, once the patient is medically stable, most stroke survivors access inpatient stroke rehabilitation at 14 days (inter-quartile range, 10–19 days) and stay at a median of 25 days (interquartile range, 19–29 days). Therefore, we evaluated the patient at 3 weeks.

A consensus has been reached regarding the treatment of deep using active anticoagulation. Anticoagulant therapy can reduce the possibility of intramuscular venous thrombosis progressing to deep venous thrombosis near the heart and can significantly improve the recanalization rate of the intermuscular vein.

Previous studies^[23]^ have suggested that patients with acute DVT should be administered therapeutic anticoagulation, strict bed rest, and lower limb braking. However, in the 2017 guidelines^[24]^ for the diagnosis and treatment of deep venous thrombosis (Third Edition), physical therapy was shown to be an effective measure to prevent the formation and recurrence of lower extremity deep venous thrombosis.

Only one factor associated with recanalization was found: the “mobility” of the patient (i.e., patients with significantly reduced mobility had a lower probability of MCVT recanalization and a higher probability of extension^[19]^). Limb immobility, whole body immobility, or neurological immobility was related to a higher risk of DVT. Patients with significantly reduced mobility had a lower probability of developing CDVT recanalisation.^[19]^ In patients receiving conservative treatment, the average incidence of proximal spreading of isolated DVTs is approximately 10%.

After rehabilitation training, the FMA score of patients was significantly improved; that is, the activity ability was significantly improved, and the recanalization rate of thrombosis and the progression of thrombosis were lower than those in the non-rehabilitation group.

The present study has several limitations. No interobserver variability was assessed for CDVT diagnosis and clot measurement, and some concern may exist regarding the homogeneity of the diagnostic procedure in the study.^[25]^ Notably, all ultrasound tests were performed by board-certified vascular medicine physicians using a standardized examination protocol, and previous studies^[26]^ have shown that compression ultrasound yields satisfactory inter-agreement. Finally, the non-rehabilitation group of patients with MCVT was small, and larger studies are required in such patients. The optimal treatment for post-stroke DVT requires high-quality randomized controlled trials.

## 5. Conclusion

The present study revealed that, on the basis of anticoagulation, rehabilitation training did not increase the thrombosis progression of MCVT and was effective in the recovery of lower limb motor function in stroke patients. This was a retrospective study. To better prevent and treat lower extremity deep venous thrombosis after stroke, large-sample randomized controlled trials should be carried out in future research to provide more scientific information for the rehabilitation of patients with thrombosis.

## Author contributions

**Data curation:** Bao-juan Cui.

**Resources:** Dong-mei Gao.

**Software:** Wen-han An.

**Supervision:** Fan-shuo Zeng.

**Writing – original draft:** Benling Liu.

**Writing – review & editing:** Laigang Huang.
